# Development and Functional Properties of Intestinal Mucus Layer in Poultry

**DOI:** 10.3389/fimmu.2021.745849

**Published:** 2021-10-04

**Authors:** Yada Duangnumsawang, Jürgen Zentek, Farshad Goodarzi Boroojeni

**Affiliations:** ^1^ Institute of Animal Nutrition, Department of Veterinary Medicine, Freie Universität Berlin, Berlin, Germany; ^2^ Faculty of Veterinary Science, Prince of Songkla University, Hatyai, Songkhla, Thailand

**Keywords:** mucin, mucus layer, goblet cell, mucosal integrity, intestine, poultry

## Abstract

Intestinal mucus plays important roles in protecting the epithelial surfaces against pathogens, supporting the colonization with commensal bacteria, maintaining an appropriate environment for digestion, as well as facilitating nutrient transport from the lumen to the underlying epithelium. The mucus layer in the poultry gut is produced and preserved by mucin-secreting goblet cells that rapidly develop and mature after hatch as a response to external stimuli including environmental factors, intestinal microbiota as well as dietary factors. The ontogenetic development of goblet cells affects the mucin composition and secretion, causing an alteration in the physicochemical properties of the mucus layer. The intestinal mucus prevents the invasion of pathogens to the epithelium by its antibacterial properties (e.g. β-defensin, lysozyme, avidin and IgA) and creates a physical barrier with the ability to protect the epithelium from pathogens. Mucosal barrier is the first line of innate defense in the gastrointestinal tract. This barrier has a selective permeability that allows small particles and nutrients passing through. The structural components and functional properties of mucins have been reviewed extensively in humans and rodents, but it seems to be neglected in poultry. This review discusses the impact of age on development of goblet cells and their mucus production with relevance for the functional characteristics of mucus layer and its protective mechanism in the chicken’s intestine. Dietary factors directly and indirectly (through modification of the gut bacteria and their metabolic activities) affect goblet cell proliferation and differentiation and can be used to manipulate mucosal integrity and dynamic. However, the mode of action and mechanisms behind these effects need to be studied further. As mucins resist to digestion processes, the sloughed mucins can be utilized by bacteria in the lower part of the gut and are considered as endogenous loss of protein and energy to animal. Hydrothermal processing of poultry feed may reduce this loss by reduction in mucus shedding into the lumen. Given the significance of this loss and the lack of precise data, this matter needs to be carefully investigated in the future and the nutritional strategies reducing this loss have to be defined better.

## Introduction

Intestinal mucus layer is the first line of defense protecting epithelium against luminal threats including mechanical forces during digestion process, enzymes and gut bacteria. The intestinal mucus also plays important roles in supporting the colonization with commensal bacteria, maintaining an appropriate environment for digestion and facilitating nutrient transport from the lumen to the underlying epithelium. The mucus layer is produced and preserved by mucin-secreting goblet cells. The present manuscript reviews the current state of knowledge about the ontogenetic development of goblet cells and the interactions between the intestinal mucus and gut microbiota as well as the mode of actions behind intestinal mucus functionality in poultry. Furthermore, it highlights dietary factors affecting goblet cell proliferation and differentiation and the consequences of these effects on mucosal integrity and dynamic in poultry.

## Goblet Cell Development

**Figure 1:**
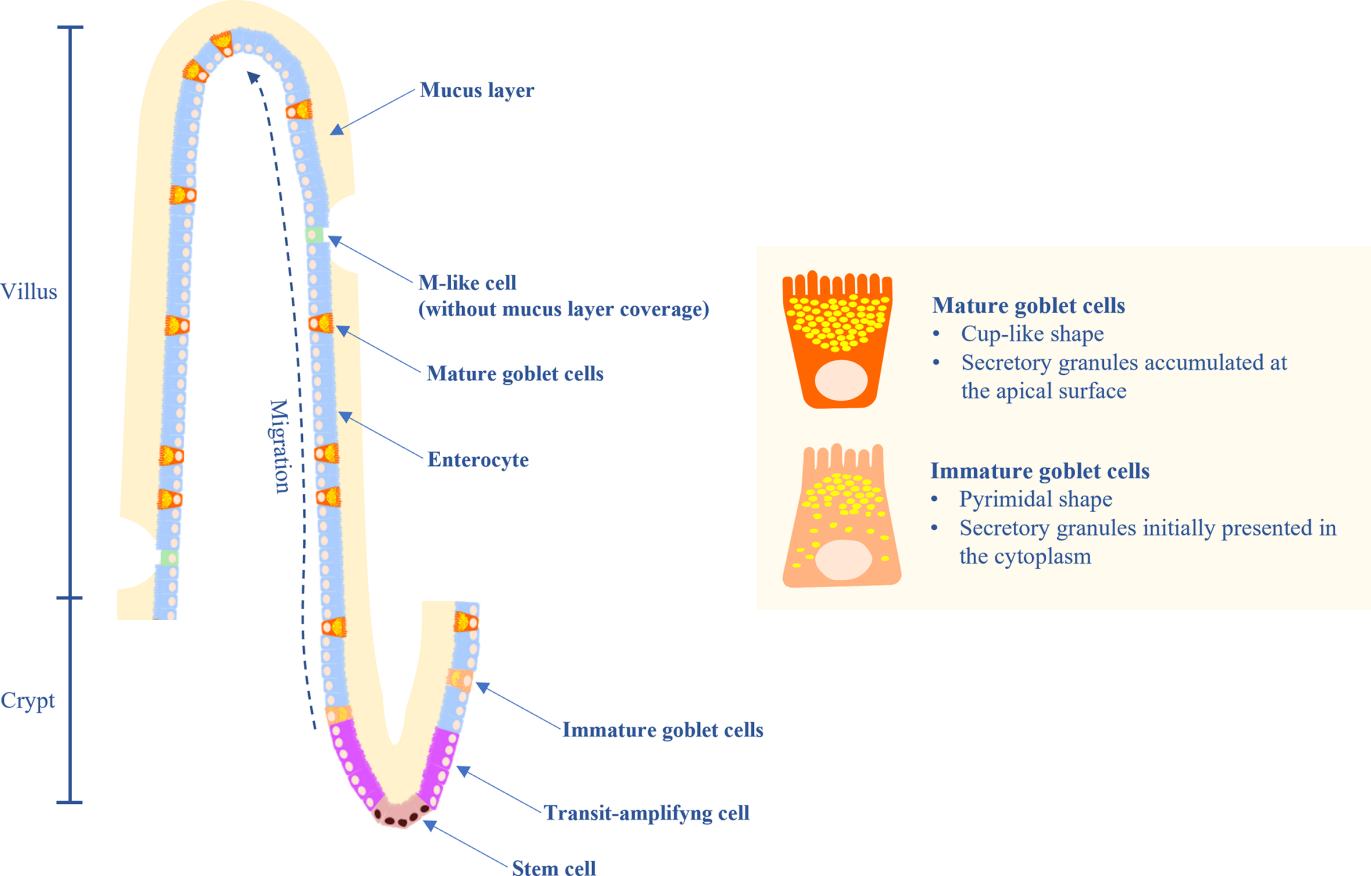
Diagram of the small intestinal epithelium highlighting the characteristics of immature and mature goblet cells (GC), GC migration, and mucus layer.

Goblet cells (GC) are highly polarized columnar epithelial cells which contain secretory granules in the cytoplasm. GC secrete mucins which provide the mucosal surfaces with a thick mucus layer lining, and separate the intestinal epithelium from the luminal cavity. The mucus layer plays important roles in maintaining the intestinal microbial balance, facilitating nutrient transport, preventing pathogen invasion and regulating the microbial–host immune response (1). GC are differentiated from the transit-amplifying cells which are the transition cells between the stem cells and differentiated cells and are located in the crypts of the small and large intestine (**Figure 1**). The intestinal crypt is a harbor of stem cells and transit-amplifying cells which are committed to produce several cell lineages including GC and enterocytes. Maturation of the GC occur along with migrating toward the villus tip, where they are undergoing the apoptosis process or being damaged and shed into the lumen. Immature GC at the crypt base are large, pyramidal in shape, and contain mucin granules. During maturation, GC become a cup-like shape accumulating more mucin granules at the apical portion, whereas the nucleus and synthetic organelles reside at the basal portion. In chicken, the migration of GC along the villus-crypt axis occurs over a duration of 2-3 days (2).

The morphology of GC in chicken can be distinguished from enterocytes at around 16.5-18 days of embryonic age (2, 3). It has been shown that, the density of GC increased by 3.3 times in the duodenum from 18 to 21 days of embryonic age, whereas this number in the jejunum and ileum increased by 4.5 and 7.1 times respectively (4). However, Uni et al. (2) found no change in GC density of the duodenum during the last 3 days of incubation (2). The GC density is variable in the jejunum and ileum during the first week of age, while it is almost constant in the duodenum (2). A marked, 1.8-fold increase in the GC density was reported in the ileum during the first 4 days of age, while no significant change was observed in the duodenum (5). The GC density in the jejunum and ileum increased by approximately 1.5 and 1.8 times from day 4 to 7 of age (6). At the end of the first week post-hatch, different developmental rates of GC along the small intestine led to an anteroposterior increasing trend in its GC density, with the lowest density for the duodenum and the highest for the ileum (2, 6). The massive increase in the intestinal GC density and activity in the first week of age seems to be due to the emerging needs of newly hatched chickens for mucus secretion and immune response, associated with their immediate expose to the surrounding environment and diet. The host-related responses after hatch seem to provide enough functioning GC to maintain mucus thickness and protect the underlying epithelia from the introduced threats in the gut lumen (6, 7).

**Figure 2:**
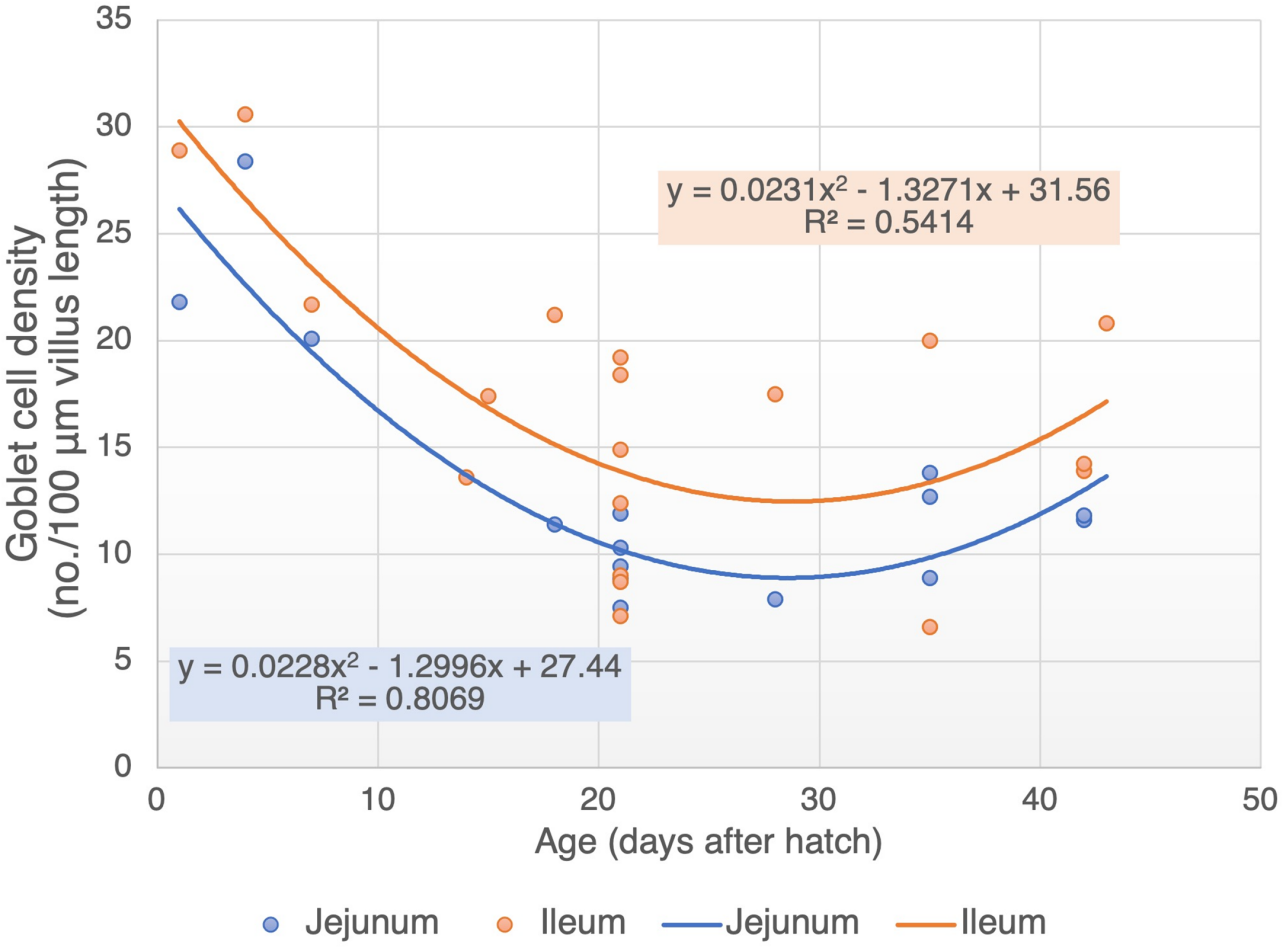
Goblet cell density in jejunum and ileum of broiler during age development (cell number per 100 µm villus length). Adapted data from references (6, 8–17).

While the GC density in the jejunum and ileum are relatively high during the first week of age, it tends to decrease afterward until the third week of life (**Figure 2**) . The GC density tends to be stable between third and fifth week of age, with an average of 10.4 and 11.3 cells/100 μm of villus length in the jejunum and ileum, respectively (**Figure 2**). Calik and Ergün (8) also reported a stable GC density in the ileum of 21 and 42 days old chickens (14.9 *vs.* 13.9 cells/100 μm villus length). Therefore, although the cell renewal of GC in chickens has not been investigated yet, it can be speculated that the GC population in the small intestine may reach maturity at three weeks of age. This speculation can be supported by the outcome of several poultry studies showing an initial decline in the cell differentiation and migration rate through a decreased mitotic activity at day 21 of age compared with days 7 and 14 (18, 19).

The predominant changes in the GC density of the jejunum and ileum during the first week of age indicates that the age-related GC development rate is region specific. The proximal part of the small intestine including duodenum is very active in digestion and absorption processes. Although GC secretion provides moisturizing and lubricant properties for epithelial cells (20), the proximal part of the intestine may prioritize the proliferation of absorptive cells over secreting cells (21), which can be associated with a lower GC density, lower mucus secretion (22) and larger GC size (23) in the duodenum compared with the jejunum and ileum. The retention time is only a few minutes in the duodenum and up to 2 hours in the ileum (24). Therefore, the lower GC density and mucus secretion in the duodenum may enhance absorptive capacity, making the duodenum accommodate to the short digesta retention time. Furthermore, the number and activity of bacteria, along with the digesta retention time, increase distally in the small intestine. The bacteria and their products are recognized by the sensor system of the intestinal and immune cells leading to an activation of the host innate defense system (25), which in turns stimulate GC differentiation *via* cellular signaling or secreted cytokines such as IL-1β, IL-4, IL-13 and L-22 (1). Thus, the anteroposterior increasing trend in the GC density and mucus secretion (in the small intestine) can be assumed as a host adaptation to enhance protective barrier against the increasing number (and activity) of gut bacteria along the small intestine (25).

## Mucin Secretion

The primary function of GC is to secrete mucins and create a protective mucus layer. Mucins are the major components in the cytoplasmic secretory granules of GC. Other proteins were also found in the GC secretion including IgA, avidin and lysozyme as well as other secretory components that play major roles in innate immunity of chicken (26). The secretion of GC is suggested to be regulated by two pathways; i) constitutive secretory pathway, and ii) regulated secretory pathway (27). The constitutive secretory pathway is a low-level continuous secretion to maintain the renewal of the intestinal mucus layer. In this baseline secretion, mucin glycoproteins are assembled and stored in membrane-bound granules which are stored within a highly organized array of microtubules and intermediate filaments called theca. The theca separates mucin granules from the rest of the cytoplasm and gives GC goblet cells a large cup-like shape (28). The constitutive secretion is dependent on cytoskeletal movement (e.g. the theca) that moves secretory granules toward the cell surface (29). This steady and unstimulated release results in maintaining the mucus layer (1). The regulatory pathway is an exocytosis of GC responding to external stimuli such as neurotransmitters (e.g. acetylcholine), cytokines, bacteria and their products including lipopolysaccharides (27). Acetylcholine, a primary parasympathetic neurotransmitter, plays a role in GC degranulation and can induce mucin secretion (30). Stimulation with acetylcholine or other cholinergic agonists such as carbachol resulted in a rapid transient increase in mucus secretion rates in the small and large intestine of mouse (31). Cytokine secretions by immune cells have been reported to stimulate GC proliferation and mucus production. For example, the secretion of IL-13 by dendritic cells and macrophages, IFN-γ by the activation of Th1 pathway and IL-4, IL-5, IL-9, and IL-13 by T helper 2 have been shown to stimulate GC proliferation and mucus production (1). The presence of bacteria which disrupts mucosal surface, has been also reported to stimulate a rapid release of stored mucin granules (32). The absence of gut bacteria in germ-free chickens led to a reduction in GC number and density as well as MUC2 mRNA expression in the small intestine compared with conventional birds. These observations confirm the stimulating impact of gut bacteria on the mucin development and secretion (33). Immediate bulk release of mucins (triggered by the regulatory pathways) captures the pathogens mechanically and inhibits them chemically with antibacterial peptides/proteins (secreted by GC and other epithelial cells), while the continuous basal secretion maintains the mucus layer during an absence of luminal or physiological stimuli.

GC in chicken has some functionalities similar to Paneth cells in mammals. In mammals, Paneth cells are restricted to crypts of the small intestine and secrete substances like lysozyme, IgA, and defensins which protect host from enteric pathogens. Among these substances, lysozyme is wildly considered as a marker for Paneth cells (34). To date, presence and location of Paneth cells in the small intestine of chickens have remained controversial. An *in situ* hybridization analysis showed that lysozyme-positive cells were specifically located at the bottom of crypts in the small intestinal of 6-month-old chickens. These detected cells also showed morphological similarities to Paneth cells in mammal (34). However, in the small intestine of 17 days old chickens, lysozyme-positive cells were only observed in the villi epithelium and were absent in the crypts (35). In a study on the duodenum of chickens, it has been shown that lysozyme-positive cells are not only found in crypts, but can also be detected along villi. It was suggested that lysozyme-containing cells located in the small intestine villi can be either GC, goblet cells, Paneth cells, or lysozyme-positive enterocytes (26).

Mucins are synthesized and readily secreted by GC at the crypt, while their compositions and secretion rate change along with cell migration. During migration of GC from the crypt, the mucin secretion and renewal rate increase (22) and the oligosaccharide chains in the mucin glycans are elongated by the addition of monosaccharides (36). The elongation of mucin glycans and higher secretion of mucin may indicate the maturation of GC along the migration toward the villus tip. In a mice study, it has been shown that, the duration of mucin synthesis is around 3-4 hours in the crypts and less than 3 hours in the villi (22). A faster mucin production and secretion by GC at the villi compared with crypts may be a physiological response to facilitate luminal mobility and digestion as well as protect the villi surface against mechanical erosion and microbial invasion (22), while the preserved mucins at the crypt can provide a further protection by a massive release in case of gut inflammation and infection (37).

A well-developed mucus layer in the gut is important for an active immunity system. Beside mucin secretion, the gut GC also participate in the immune responses by secretion of various substances acting as antibacterial agents. GC in the duodenum and cecum of broilers were shown to store avidin, lysozyme and other secretory components (26). Avidin was found to be an acute phase protein which is expressed in the intestine during gut injury and inflammation (38) and involves in restoration of a damaged intestinal tissue (39). Secretory components like cleaved fragments of pIgR, have neutralizing properties against pathogen-associated molecules and act as antibacterial substances (26). Lysozyme plays an important role in activating innate immunity and recruiting of leukocytes (26). Immune protection of the gut in early life stage depends on provision of maternal antibodies including IgA, IgG and IgM which can be delivered *via* colostrum and milk in most of mammals (40). However, industrial avian species have no direct contact with parents after the egg is laid; hence, the only source to supply maternal antibody is the egg itself. Maternally derived IgA was found in the GC and epithelial apical surface of newly hatched chickens. Maternally derived IgY (the avian counterpart to mammalian IgG) was observed in the intestinal vessels at the day of hatch. Both Ig appeared later (7-28 days of age) in the plasma cells located in the lamina propria of the small intestine (41). The GC seems to act as a reservoir for maternal IgA antibodies prior to hatch which are slowly secreted along with mucin, thus extend the protection until maturation of the endogenous IgA response (41). During the first week after hatch the maternal IgA in chickens decreases gradually and the maturing antibody secreting cells subsequently take over the immunological protection (7).

## Mucin Characteristics and Goblet Cells Categorization

**Figure 3:**
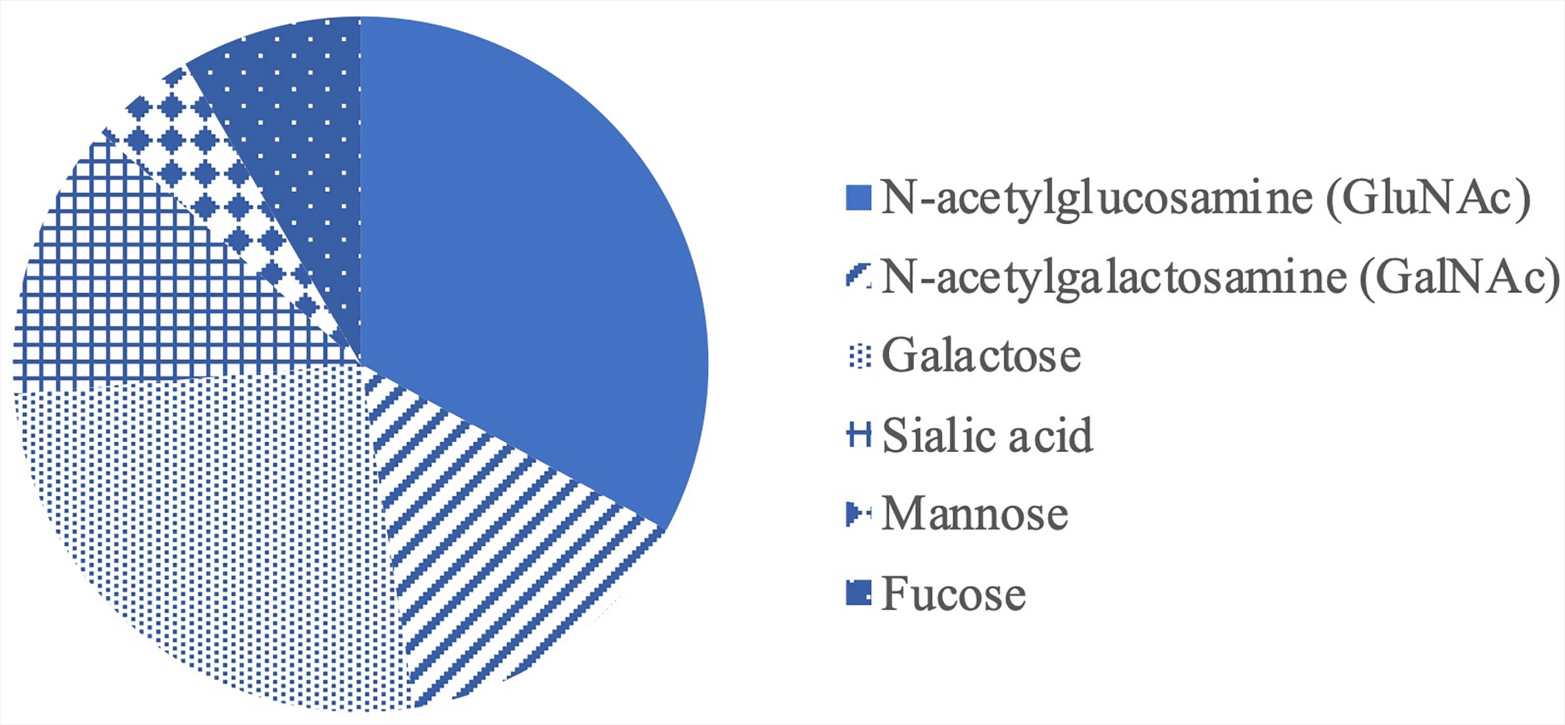
Proportion of mucin monosaccharides in the small intestine of broiler (46, 47).

Structurally, mucins are large glycoproteins characterized by heavily *O*-glycosylated polypeptides that usually composed of tandem repeats rich in proline, threonine and/or serine sequence (PTS domain). The hydroxyl group of threonine and serine is necessary for ester linkages between the amino acid backbone and carbohydrate groups (42). A dense array of *O*-linked carbohydrates in mucins confers charge to them, giving them the ability to interact with other surrounding molecules such as nutrients and regulate the diffusion of nutrients within the mucus layer. Mucin *O*-glycans account for over 80% of the total mucin molecule by mass and support rigidity of the mucin structure, contributing to specific physical and biological properties essential for their protective functions (43). For instance, the chicken MUC2 mucin comprises 3,697 amino acids (44) and >100 different *O*glycan structures linked to the mucin protein core (45). Mucin *O*-glycans typically contain several types of sugars (**Figure 3**) including N-acetylglucosamine (GluNac), N- acetylgalactosamine (GalNac), galactose, sialic acid, fucose and mannose (46–48). The glycan biosynthesis begins by the transfer of one sugar to the protein, followed by the addition of more monosaccharides one by one (32). In chicken, *O*-glycans are predominantly composed of GlcNAc, galactose and GalNAc at approximately 37.0, 27.4 and 13.4%, respectively, while the remaining includes sialic acid, fucose and mannose (46). Further structural diversity is obtained through modifications of the saccharides e.g. phosphorylation, sulfation, and acetylation (32). The most common terminal end of glycans in the small and large intestine of chicken contains sulfated and sialylated groups which account for the polyanionic or negative charges of mucins (49). The complexity of mucin carbohydrate structures is regulated by glycosylation within the Golgi apparatus of the GC. The pattern of monosaccharide sequence in mucins varies between individual GC, intestinal location and species (32).

Depending on the ionic charge of the mucin molecule, mucins can be differentiated (**Table 1**) as neutral or acidic (50). The *O*-glycans containing terminal residues of sialic acid and sulfated group represent anionic charge, while other terminal groups such as fucose, hexose and galactose result in neutral charge. The negatively charged mucins are likely to resist mucin degradation by bacterial enzymes therefore; mucin charge is an important factor in determining host defense with particular regard to interactions with microorganisms present in the gut. It is noteworthy that the *O*-glycosylation occurring in the GC is usually very heterogeneous and not only a single type of mucin is produced in an individual GC (50). Based on the sum of ionic charge of secreted mucins, GC can also be categorized into two types; 1) neutral GC predominated by neutral charged mucins and 2) acidic GC predominated by anionic charged mucins (**Table 1**). By using common microscopic observations in histological studies, staining of secretory granules in GC represents a color as the result of a chemical reaction between dyes and the terminal groups of mucins. The anionic mucins can be detected by cationic dye including Alcian blue (AB) and mucicarmine resulting in blue and red in color, respectively (51). In contrast, the neutral mucins can be recognized by the reaction between Periodic acid -Schiff (PAS) reagent and aldehyde groups of the monosaccharide units, resulting in magenta color (51). GC containing almost similar amount of acidic and neutral mucins appear in bluish purple color and are called mixed GC (52). In chicken, the mixed GC are commonly observed along the small intestine (53, 54). Most of the studies conducted on chicken have used both AB and PAS solutions for mucin determination in the gut tissue, individually or together (combined staining method). The individual staining method uses only one solution at the time for each tissue sample (determining acidic and neutral GC in separate tissue samples), while the combined staining method uses both solutions in one process (determining acidic and neutral GC in one tissue sample). Given the fact that one GC can produce more than one type of mucin (2), overestimation of acidic and neutral GC number may be more likely to happen by using the individual staining method. Therefore, the histochemical technique of “combined staining method” may be more accurate to define the GC types.

**Table 1:** Characteristics of neutral and acidic goblet cells ^a^.

	Neutral goblet cells	Acidic goblet cells
**Mucin structure**	Large glycoproteins containing extensive amounts of oligosaccharide chains attached to protein core	
**Mucin *O*-glycan composition**	Heterogenous arrangement of monosaccharides including GluNAc, GalNAc, galactose, fucose, sialic acid and mannose	
**Terminal end of *O*-glycan**	Predominant in neutral charged monosaccharides e.g. GluNAc, GalNAc, galactose, fucose and mannose	Predominant in negatively charged monosaccharides e.g. sialic acid and/or sulfated groups
**The overall charge of mucins**	Neutral charge	Negative charge

a references (49–52).

Mucins are also broadly grouped into transmembrane and secretory mucins based on their biosynthesis and secretion. The secretory mucins are initially formed by mucin polymers and packed into secretory granules prior secretion (55). Upon mucin granule exocytosis, the secretory mucins are hydrated and expanded massively, forming a gel-like structure and creating a mucus layer over the intestinal epithelium. The transmembrane mucins are characterized by a single transmembrane protein incorporated into the plasma membrane, a cytoplasmic tail (signaling domain) and the extracellular part of a highly glycosylated mucin domain. These mucins are retained at the apical cell surface of GC (55). The transmembrane mucins do not form gel-like structure but rather serve as anchors for the secretory mucins. However, the strict classification of both mucin types is complicated by their dual occurrence of membrane attachment and detachment; the transmembrane mucins can be forced to detach from the cell surface by mechanical stimuli (56), while the secretory mucins temporarily attached to the apical surface of GC prior the cleavage by proteolytic enzymes (1). Proteolytic enzymes such as Meprin β are likely to cleavage the specific site at the anchor point between secretory mucins and cell membrane. This cleavage is found to be activated through bacterial contact or microbial signaling to the enterocytes, indicating that the presence of gut bacteria may be a key mechanism for secretory mucin detachment and release into the intestinal lumen (57). It has been observed that Meprin β was deficient in the small intestine of germ free mice resulting in less viscous mucus layer, thus more adhesive and attached mucus to the cell membrane (57).

Mucins are encoded by mucin genes, represented as MUC followed by a number that reflects the order in which the particular mucin gene was discovered. A mucin gene is translated into a protein core which is further decorated with extensive glycosylation. The discovered MUC genes in chicken are still limited compared with human (**Table 2**). Different types of mucin are present throughout the gastrointestinal tract in specific locations. Each type plays a different role in maintaining homeostasis and protecting intestinal epithelium in different parts of the gut. In chicken, the MUC2 is the major component of mucus in the small and large intestine but it is weakly expressed in the crop (44), whereas MUC5ac is specifically expressed in the proventriculus and only weakly expressed in the small intestine (6, 45). The composition and sequence of amino acids derived by specific MUC genes have a role in dimerization through covalent and noncovalent cross-links and their subsequent polymerization to form multimers (61, 62). The structure conformation of different MUC type mucins (such as MUC2 *vs.* MUC5ac) was found to differ in cross-linking between mucin dimers, which may determine their functionality in regard to mucus permeability (61) and viscosity (62) in different parts of the gastrointestinal tract. The tissue specific expression of MUC genes seems to be depending on the physicochemical properties of the mucins and their required functionality in maintaining mucosal integrity in that specific part of the gut.

**Table 2:** Mucin genes expressed in the small and large intestine of human and chicken.

Mucin types	Human ^a^		Chicken ^b^	
	Small intestine	Large intestine	Small intestine	Large intestine
**Transmembrane mucins**	MUC1	MUC1	MUC4	n/a
	MUC3	MUC3	MUC13	
	MUC4	MUC4		
	MUC12	MUC12		
	MUC13	MUC13		
	MUC15	MUC15		
	MUC16	MUC17		
	MUC17			
**Secretory mucins**	MUC2	MUC2	MUC2	MUC2
	MUC5ac	MUC5b	MUC5ac	MUC6
	MUC6			

n/a, no available data.

a references (56, 58).

b references (59, 60).

## Development of Acidic and Neutral Mucins

The physiological relevance of distinct mucin types is not yet well understood but it has been suggested to be associated with their protective properties. The distribution of mucin types in the GC is regulated by glycosylation process, which can be affected by host (e.g. inflammatory markers, hormones and neurotransmitters) and external (e.g. commensal bacteria, pathogens, pre/probiotics and nutrients in the diet) factors (20). It is known that many bacteria in the gut produce glycosidases or proteases to degrade mucus. The terminal ends of *O*-glycan with *O*-acetylated sialic acid (sialomucins) or sulfated group (sulfomucins) have been found to play a key role to protect mucin chains from degradation by bacterial enzymes (like proteases and glycosidases) and proteolytic host enzymes (52, 63). Previous studies on the chicken’s intestinal mucosal layer and mucin subtypes indicate that during late embryonic development, the produced mucins are more of the acidic subtype than neutral subtype (2, 4, 64). It seems that acidic mucins production prevails before hatch and then neutral mucins are more produced after hatch (6). In newly hatched chicks, the density of acidic and neutral GC was increased during the first week of age in the small intestine, especially in the jejunum and ileum. This increase was suggested to be due to the bacterial colonization in the gut and presence of dietary components (6, 7). The dominance of acidic mucin production by GC right before hatch could be a host adaptation to proteolytic host enzymes (to use yolk sac) and prepare the chicken for exposure to bacterial proteases and glycosidases right after hatch (2).

The development of sialomucins and sulfomucins from late embryonic stage to post-hatch is shown in the **Table 3**. Sulfomucins appear initially as early as 18 days of embryonic age (4), while mucins containing sialic acids appear later at 21 days of embryonic age and considerably increase after hatch, especially during the first week of age (4, 6). The high amount of sulfomucins presented at embryonic stage may be due to the immaturity of GC (because of the low number and activity of gut bacteria) (6). No exposure of hatching eggs to the caecal content of the layers caused a reduction in the number of both neutral and acidic GC with the absence of sialomucins during the first 7 days of age, compared with the chickens hatched from conventional eggs (33). The presence of intestinal bacteria could induce GC maturation and increase mucus production which could associate with increasing amount of sialomucins after hatch (6). It was found that GC initially contain higher amount of sulfomucins and lower amount sialomucins, but as they mature and migrate toward the villus tip, the mucins are increasingly sialylated (63). This can indicate the importance of bacterial exposure for GC differentiation and maturation (33).

**Table 3:** Percentage of acidic goblet cell number in the jejunum and ileum of broiler at different ages.

Intestinal part	Age ^a^	Percentage of acidic goblet cell number ^b^			Reference
		Sialomucin	Sulfomucin	Intermediate	
Jejunum	E18	–	100%	–	(4)
	E21	20%	56%	24%	(4)
	D1	–	100%	–	(6)
	D4	31%	38%	38%	(6)
	D7	18%	41%	41%	(6)
	D18	34%	49%	17%	(65)
	D18	30%	54%	16%	(10)
Ileum	E18	–	100%	–	(4)
	E21	37%	18%	45%	(4)
	D1	–	100%	–	(6)
	D4	28%	39%	39%	(6)
	D7	28%	39%	33%	(6)
	D18	33%	47%	20%	(10)

a Age was reported as days of embryo age (E) or chicken age (D).

b Acidic goblet cell was determined by the combined staining Alcian Blue/High Iron Diamine (AB/HID) method. AB-positive goblet cells (blue) are categorized as sialomucin, HID-positive goblet cells (brown) are categorized as sulfomucin, and goblet cells that are positive to both AB and HID stains (brown-black color) are called intermediate. The percentage of each type is relative to the total number of acidic goblet cells (the sum of sialomucin, sulfomucin and intermediate).

## Mucus Layer and Its Thickness

The thickness of mucus layer is a result of dynamic balance between secretion rate by GC and destruction rate through mechanical shear and enzymatic degradation (66). During a normal physiological condition, the mucus thickness is determined by the basal secretion which involves the continuous production and release of mucins into the gut lumen as previously discussed. Two methods have been applied to measure mucus thickness from the basal secretion. One is by using cryostat-sectioned tissue stained with histochemical staining (CRHS) and the other is by using anesthetized animal (*in vivo)*. The CRHS method is a simple method that can be used for a wide range of tissue types and can show the characterization of normal mucus layer. The *in vivo* method provides information about the dynamic of mucus secretion under a real physiological regulation (67).

By microscopic observation, it has been shown that the intestinal mucus consists of 2 layers; i) a thin inner layer which is strictly attached to the epithelial membrane ii) a thick outer layer which is loose, non-attached and forming viscous gel between the lumen and the thin inner layer (68). It can be expected that the inner layer contains transmembrane mucins because it was hardly removed by mucolytic agents (69), while the outer layer may contain secretory mucins which form a viscous mucus layer (1). In human and rodent, mucus in the small and large intestine have both layers, while the inner layer of the small intestine is thinner than the large intestine. However, several studies in both species showed that the inner layer was absent in the small intestine, especially in the duodenum and jejunum (56, 70). It seems that generally, the thickness of mucus is higher in the distal part (caecum > ileum > duodenum and jejunum) of the intestine (68), which may be explained by the digestive and protective functions of mucus and the fact that, gut bacterial number and activity increase from the proximal part of the small intestine to the distal part of the gut. Few studies measured the mucus thickness in chicken by the histological method. The average of mucus thickness in 42 days old broilers ranged from 14.9 μm in the duodenum to 18.6 μm in the ileum (46, 47). In a CRHS study using anesthetized rats, the basal mucus secretion rate was the highest in the colon (3.9-5.2 μm/min), while the secretion in the small intestine ranged from 1.9 to 4.7 μm/min, with the highest rate in the ileum compared with duodenum and jejunum (68). Furthermore, it has been shown that by removing mucus layer with a suction probe, the inner mucus layer remained attached to the mucosal surface, while the outer layer in all parts of the gut was easily removed by suction collection. The mucus secretion was immediately stimulated after the mucus suction, with a lower secretion rate in the small intestine and a higher rate in the colon compared with those prior mucus removal (68). Thus, the mucus layer at the lower part of the gut seems to be better maintained resulting in a thicker mucus layer covering the epithelial surface of the colon.

## Nutrient Transport Through Mucus Layer

The intestinal mucus must provide a robust barrier that traps and immobilizes potentially hazardous compounds such as pathogens, while allowing the passage of nutrients to the epithelial surfaces. These properties are particularly important in the small intestine, where the mucus layer is the thinnest in the gut and the nutrients absorption needs to be highest. A thinner mucus layer in the small intestine could facilitate nutrient absorption, whereas a thicker mucus layer in the colon must be a barrier to the dense bacterial population (71). Since more than 90% of the total nutrients absorption including carbohydrates, proteins and lipids occur in the small intestine (72), the mucus properties involved in nutrient diffusion are of interest. It has been reviewed by Leal et al. (72) that physiochemical properties of mucus like pore size, viscoelasticity, pH, ionic strength, and net charge of mucus layer and mucin polymers can alter the transportation of molecules (**Table 4**) (72). They suggested that these factors regulate permeability of mucus layer which not only restrict the diffusion of bacteria and macromolecules but selectively, allow absorption of nutrients (72).

**Table 4:** Physiochemical characteristics of mucus layer affect nutrient transportation ^a^.

Characteristics	Impact on nutrient transportation
Pore size	Size-filtering property
Viscoelasticity	Lower mucus viscosity provides a higher permeability for diffusing molecules
pH	Higher pH increases electrostatic interaction between mucins which enhances selective permeability of charged particles
Ionic strength	Higher ion concentration enhances permeability of positively charged molecules
Net charge	Attractive or repulsive forces between diffusing molecules and the mucus

a references (72–76).

The net-like structure of mucus layer creates pores which allow only small molecules from the lumen pass through the mucus layer and restrict the flow of large molecules including polysaccharides and polypeptides (1). Limited studies evaluated the pore size of the intestinal mucus but it is known that the mucin network expands 2-3 times in volume when moving from the inner layer to the outer one (1). Several studies reported that particle size ranged from 0.5-2 µm in diameter could diffuse through the outer mucus layer of the jejunum (73) and ileum (70), while the inner mucus layer are sufficiently small (< 0.5 µm diameter) to hinder penetration of bacteria or beads (1).

The viscosity of mucus layer is attributed to the capacity of mucin monomers to form polymeric structures. Only the secretory mucins are properly assembled into a disulfide-bridged covalent network, giving mucus its viscous properties, while transmembrane mucins are monomers that are integrated into membranes and do not form viscous gels. The viscosity of the mucus ranged from 1 to 30,000 millipascal second along the villus surface of the pig’s small intestine, with a numerically higher mean viscosity at the inter-villus space (the space between the villi) compared with the villus tip (73). In general, a low viscous mucus provides a higher permeability for diffusing molecules (74), thus the diverse viscosity at different part of the villi may indicate the preferential area for nutrient diffusion. However, diffusion of particles through mucus layer was greater at the inter-villus space compared with villus tip due to a hindrance of the apoptotic cells that shed into the mucus (73).

Different components in the mucus such as water, mucins, globular proteins, salts, DNA, lipids, cells and cellular debris are stabilized by covalent and noncovalent interactions including hydrophobic, electrostatic and hydrogen bonds (72). These binding interactions are the main factors that contribute to viscoelasticity and permeability of a mucus layer (72). Generally, charged groups of the mucins can interact with charged particles and immobilize them through the mucus (74). Peptides which contain both basic and acidic amino acids have both positive and negative charges simultaneously. It was found that when positive and negative charges were both present on a peptide, the diffusion through gastric mucins was higher than the isolated charged peptides (75). The interaction between charged particles and mucins also rely upon the intestine’s pH as well as ionic strength of the intestinal mucus (74). Lowering the mucus pH altered mucus conformation by promoting the exposure of hydrophobic domains of the mucins, decreasing repulsive forces between mucins and increasing mucus viscosity (72). Thus, a reduced electrostatic interaction between mucins enhances selective permeability against charged particles compared with neutral particles (72). In porcine gastric mucins with pH adjustment to 3, the positive and negative charged polyethylene glycols (PEG) were less mobile leading to low diffusion, while both charged and neutral particles diffused almost freely in mucus at pH 7 (74). The pH limitation for nutrient diffusion through mucus layer may be lesser in the distal part of the small intestine compared with the proximal part because of the fact that in chicken, pH increases from the duodenum (5.0-6.0) to the jejunum (6.5-7.0) and ileum (7.0-7.5) (76).

Changes in ionic strength cause shrinkage or swelling of mucus and, thus, significantly alter mucus viscoelasticity (77). The strength of the attractive or repulsive forces between mucin molecules depends on the ion content in the mucus layer including sodium, chloride, potassium and calcium ions (74, 78). In general, increases in ion concentration correlate with a decrease in the viscosity of mucus (77). The investigation of ionic strength in the intestinal mucus layer of chicken has not been yet explored. Using porcine gastric mucus (*ex vivo*), it was demonstrated that the mobility of positively charged particles was considerably increased at high ionic strength (500 mM NaCl, pH 3) compared with low ionic strength conditions (20 mM NaCl, pH 3), while neutral particles diffusivity remained unaffected by changes in the ionic strength (74). Similarly, increasing the ionic strength (5-200 mM NaCl, pH 7) of porcine gastric mucus accelerated the transport rate of cationic peptides (lysine residue), while the anionic peptides (glutamic acid residue) maintained a high diffusion at various ionic strengths (75). Therefore, it can be speculated that that high ionic strength of mucus layer in a neutral pH condition, which usually occurs in the lower part of the small intestine (compared with the proximal part of the gut), may lead to a higher permeability of positively charged molecules in this part of the gut.

## Protective Mucus Layer Against Gut Bacteria

The intestinal mucus layer provides a protective shield for epithelium against gut microbiota which begins to colonize within an hour after hatch (79). The bacterial colonization was initially observed in the cecum, possibly because of yolk sac utilization and absorption effect, which was dominated by facultative aerobes such as *Enterobacteriaceae* and *Streptococcus* spp. and then spread throughout the gastrointestinal tract within 24 hours (79, 80). It has been reported that the bacterial concentration increases distally along the small intestine due to increasing luminal pH and retention time in the distal ileum compared with the duodenum (79). Approximately one third of the commensal bacteria is comprised of genes involved in carbohydrate digestion and many bacteria have specialized genes for degrading different type of complex carbohydrates such as non-starch polysaccharides (81). In a normal condition, gut bacteria locate only in the outer mucus layer where they can degrade mucin glycans or proteins and utilize them as energy source for colonization, while the inner layer is relatively impermeable for bacteria (82). However, when the mucosal barrier function is disrupted, the mucus becomes more permeable and a higher number of bacteria can be found in the inner layers (82). A more invasive bacteria including pathogens may extensively degrade mucins and compete with the gut microflora for mucin-derived nutrients, establishing their colonization and epithelial attack.

The homeostasis of gut bacteria in chicken can be affected by mucin (MUC) types, *O*-glycan composition (extent of glycosylation and oligomerization of mucin), and the mucus layers characteristics (inner and outer mucus layer thickness) (50, 83). Mucin types and *O*-glycan composition affect physicochemical properties of mucins and the effectiveness of bacteria for reaching epithelial cells by degradation of mucins (50). Several mechanisms for the intestinal mucus were reported which prevent the invasion of pathogens, while maintaining a homeostatic microbial population. The continuous secretion of mucus pushes the pathogens away from the enterocytes and flushes them out distally with peristaltic moves (73). Moreover, antibacterial peptides and proteins within the mucus prevent a direct access of bacteria to the epithelial surface. In chickens, antibacterial compounds in GC secretion including β-defensin, lysozyme, avidin, IgA, and free secretory component (a glycoprotein that binds and transports the secretory immunoglobulins) were found as responses to both gram positive and negative bacteria (26). The continuous secretion of these peptides and proteins creates an antibacterial gradient within the mucus layer with an increasing antibacterial activity from the lumen to the cell surface, creating stricter protection at a closer area to the epithelium (56). Due to a shielding or charge repulsive effect of the anionic glycans, the interaction between mucins and pathogens could also slow down their penetration. A high abundance of sulfated and sialylated mucins could reduce the adhesion and penetration ability of *Campylobacter jejuni* through intestinal mucus of chicken and protects the mucins from degradation by bacterial glycosidases (84). The protective properties of mucus may also rely on mucin subtypes (e.g. MUC2 and MUC5ac) through the interaction between protein domains which as discussed, can determine the permeability of the mucus layer (25, 61).

Although the small intestine contains lower bacterial population than the lower gut, it may be a better target for pathogenic bacteria due to its thin and patchy distributed mucus layer. Furthermore, a particular area in the small intestine lacks of mucus coverage (**Figure 1**). This area composes of M-like cells overlying on the lymphoid tissues of the digestive tract and bursa of Fabricius (32). The M-like cells act as sentinel cells which transport endocytosed microorganisms and other antigenic substances into the underlying lymphoid structures and initiates immune response. However, some bacteria including *Salmonella* Typhimurium*, Shigella flexneri, Yersinia enterocolitica* and *Vibrio cholerae* take advantage of the low protective barrier of this area and invade the epithelial cells (32).

Intestinal mucus not only serves as a protective layer but also accommodates the colonization of bacteria by providing i) ligands for bacterial adhesion, and ii) nutrient sources for selective bacterial community that contains mucin degrading enzymes or receives degraded mucin saccharides from the others (83). The colonization ability of bacteria depends on the bacterial attachment, bacterial enzymes for mucin degradation and utilization capacity of them for mucin-derived carbohydrate. These mechanisms were extensively reviewed by Sicard et al. (83). The bacterial adhesion to mucins is believed to initiate the colonization process which involves one or more mechanisms including Van der Waals forces, electrostatic interaction, and hydrophobic forces (85). Bacteria frequently use different strategies like cell-surface proteins, pili, fimbriae and flagella to bind to mucins (83). Some commensal bacteria including lactic acid bacteria occupy mucus binding proteins and pili to adhere to mucin oligosaccharide (e.g. mannose), while other adhesion strategies were observed for pathogenic bacteria. For example, *C. jejuni* uses the carbohydrate-lectin, flagella subunit proteins and major outer membrane proteins to adhere to mucins (83).

Mucolytic bacteria possess specific enzymatic activity necessary to degrade glycan chains and facilitate their colonization. Their enzymes include neuraminidases/sialidases, fucosidases, exo- and endo-β-N-acetylglucosaminidases, β-galactosidases, α-N-acetylglucosaminidase, and α-N-acetylgalactosaminidases (86). The members of mucolytic bacteria are groups from both commensal (such as *Bifidobacterium bifidum, Bacteroides fragilis, Akkermansia muciniphila)* and pathogenic bacteria (such as *Clostridium perfringens, Salmonella* Typhimurium*, Vibrio cholerae, Enterococcus faecalis).* These bacteria compete for mucin-derived nutrients (86). Some of the intestinal pathogens have developed strategies to win this competition with commensal microflora for nutrients. It has been found that *Clostridium perfringens* have developed an ability to secrete a wide range of glycosidases with broader substrate specificity (87). *Clostridium perfringens* has shown a wide range of enzymatic activity in cleaving the terminal residues (exoglycosidases) and inner parts of sugar chains (endoglycosidases) including α-L-fucosidases, endo-α-GalNAcase and sulfatases, which promote adherence to the mucin carbohydrate receptors and mucin degradation (43). Therefore, a broader range of substrates is available for *Clostridium perfringens* which increases their chance to win the competition and cause necrotic enteritis in poultry. Another set of gut bacteria without mucolytic activity can also take advantage of released saccharides by scavenging the liberated sugars, leading to an increased in their colonization in the gut. For example, the presence of *B. thetaiotaomicron* or *B. fragilis* that produce sialidases enabled the expansion of sialic acid-scavenging bacteria including *E.coli* (88).

It has been shown that the common modification of terminal glycans of human mucins including sulfation and sialylation, increases resistance to microbial glycosidases and thus, the protective barrier is thought to remain more intact (6, 86). The 5increase in anionic (sialytated, sulfated and sialytated-sulfated) glycans was also reported in chicken and other avian species as a strategy to provide a charge-repulsion effect between mucins and maintain relatively low pH of mucins, aiming to create strong protection for mucins against bacterial degradation (84). In chicken, the *O*-glycans were abundantly sulfated and sialylated, with 33% and 23% in the jejunum and 34% and 29% in the cecum, respectively, while the remaining was neutral glycans (40% and 26%) and sulfo-sialylated intermediate (4% and 11%) in the respective locations (84). However, some bacteria possess strong sialidase and/or sulfatase activity. For example, *Ruminococcus gnavus* and *Akkermansia muciniphila* have sialidase activity, while *Prevotella* spp., *Bifidobacterium* spp. and *Helicobacter pylori* have sulfatase activity (86). Some bacteria produce both enzymes such as *Bacteroides fragilis, B. thetaiotaomicron* and *Bifidobacterium bifidum* (86, 88). Among bacteria with sialidase activity, the released sialic acid can be only utilized by some groups like *R. gnavus* and *B. fragilis* since they are the ones encoding specific genes responsible for sialic acid metabolism. On the other hand, *Salmonella* Typhimurium and *Clostridioides difficile* are able to utilize sialic acid but lack the sialidase enzyme, and thus, they rely on other sialidase-producing organisms to acquire this potential nutrient source (86, 88).

## Endogenous Loss of Mucus Layer

Mucus degradation generally occurs due to physical disruption by mechanical shear forces of peristalsis and enzymatic cleavage by bacteria, after which the mucus is transported with the intestinal content and excreted (20). Broilers have a short intestinal retention time to support a high feed intake despite the limitations of the digestive tract volume. The average retention time in broilers is 2.9 and 5.7 hours for the small intestine and total tract, respectively (24). Furthermore, chicken has a unique mechanism of intestinal reflux that propel liquid material from the proximal ileum or cloaca ascendingly to as far as the duodenum and gizzard in order to enhance digestibility of major nutrients such as starch (89). The high passage rate and frequent intestinal movement in chicken may contribute more to loss of the mucus layer compared with other species. It is noteworthy that the outer mucus layer is more loose and prone to be propelled with digesta transportation compared with the inner layer (68). Hydrothermally processed diets such as pelleted, extruded and expanded, feed may provide low mechanical shear force due to the rapid disintegration once moistened (90). Moreover, these forms of feed have higher starch digestibility compared with the non-processed one (91), reducing the need for frequent intestinal reflux to enhance digestibility of starch. It has been shown that pelleted feed increased villus height, decreased crypt depth and GC density in the small intestine of broilers compared with mash feed (92). It can be assumed that higher GC density in broilers fed mash feed is an adaptive response to increase mucin secretion and replace the part of the mucins which has been lost due to enzymatic hydrolysis, mechanical shear forces or intestinal refluxes. Thus, hydrothermal processing of poultry feed may reduce mucus shedding into the lumen.

To our best knowledge, the direct effect of particle size (fine/coarse) on mucins secretion in poultry has not been investigated yet. The coarse and fine feed are usually characterized by discrete mean particle size (dMEAN) based on dry sieving analysis; dMEAN above and below 1.8 mm are defined as coarse and fine particle sizes, respectively (93). It is generally believed that fine particles increased the accessibility of digestive enzymes to the substrate and enhance nutrient digestibility (90). However, some studies have shown that reduction in particle size of cereals (e.g. corn) did not affect ileal digestibility of amino acids that are accounted for more than 50% of the amino acid content of each type of mucin i.e. glutamic acid, aspartic acid, proline, threonine and serine (93) as well as crude protein (94, 95). Other studies showed a lower ileal digestibility of crude protein in fine corn compared with coarse corn (96, 97). It was suggested that finely ground particles may cause gut functional impairment due to a faster passage rate (98), while coarse particles reduce passage rate and enhance gizzard activity which may subsequently stimulate more bile acid and pancreatic secretion and also improve digestibility of nutrients like starch (91, 99). The dietary replacement of fine corn (2.4 mm) with coarse corn (7.16 mm) by 25% or 50% in pelleted feed increased digesta retention time, gizzard weight, and apparent ileal digestibility of energy and nitrogen, while the digesta pH seemed to be decreased in the proventriculus (100). The lower pH of the digesta entering duodenum may enhance mucus degradation by enzymatic hydrolysis (101) and may increase mucus secretion in response to prevent epithelial damage (102). In terms of nutrients absorption, the lower pH of the digesta entering duodenum can increase mucosal viscosity which as discussed, subsequently may cause lower permeability for certain nutrients. Furthermore, replacing fine particles with coarse ones could increase intestinal muscle (tensile strength) activity (100) which on one hand, may increase mechanical shear force between luminal materials and intestinal mucosa, enhancing mucus loss into the lumen and on the other hand, increases retention time of digesta leading to a higher nutrient digestibility and subsequently less intestinal reflux and loss of the mucus layer.

Mucins are high molecular mass (2 x 10^6^ Da) of heavily *O*-glycosylated polypeptides which resist to digestive processes resulting in a significant fraction of endogenous losses (103). The endogenous loss of mucin carbohydrates and amino acids are commonly determined at the end of ileum instead of total tract because of the variable and modifying effects of the hindgut microbiome on nutrient utilization (104). The quantification of mucins in ileal digesta is therefore often undertaken using mucin carbohydrate as markers. The measurement of sialic acid in ileal digesta or excreta is used to estimate the total mucins secreted into the lumen of chicken (105), while other components including fucose, galactose, glucosamine and galactose are more commonly used in mammals (106). In chicken, sialic acid concentration in the ileal digesta was reported to be between 31.7 to 171 mg/100 g dry ileal digesta (107, 108). The observed variation in the studies may be due to the different methods in sample preparation. The mucin extraction from digesta may give a lower sialic acid concentration compared with measuring it in an intact digesta (as-is). The mucin extraction method may provide only mucin-bound sialic acids and exclude bacteria-derived sialic acids including peptidoglycans and lipopolysaccharides (109). Approximately 74% of sialic acid content in the ileal digesta of pig did not bind to the mucin subunits, while the remaining (26%) was found as a mucin-bound form (110), thus the considerable amount of non-mucin-derived sialic acids may affect the overall sialic acid concentration in the ileal digesta. The variation in sialic acid concentration was also observed in the total tract excreta ranging from 76.5 to 148 mg/100 g dry excreta (111–113). Therefore, sialic acid content of the intact digesta cannot be a reliable representative for mucus loss since a major part of the measured sialic acid might be originated from bacterial cell surface (104). To gain more realistic data on the mucin loss, a further step to achieve purified crude mucin prior to carbohydrate quantification may be needed.

The endogenous proteins entering the digestive tract are predominantly originated from various digestive enzymes, mucoprotein and desquamated enterocytes. These proteins are mainly reabsorbed in the small intestine. The basal loss of endogenous amino acids in broilers (measured at the end of the ileum) was ranged from 3.08 g/kg dry matter intake (DMI) for a protein-free diet to 8.81 g/kg DMI for a casein diet (104). It is assumed that highly purified and digestible proteins such as casein are completely digested and absorbed in the small intestine of broilers (114) therefore, the detected amino acids at the end of the ileum of broilers fed by casein diet should be of endogenous origin. The remaining of unabsorbed endogenous proteins that passes beyond the ileum is considered as a loss of protein and energy to animal. The mucin polypeptides are relatively resistant to endogenous protease since the central mucin domains are protected by high density of *O*-glycosylation and animals do not secrete enzymes that can degrade the *O*-glycans (115). Therefore, increasing mucin secretion into the gastrointestinal tract spontaneously increases endogenous protein loss. In chicken, the amino acids of mucins are mainly composed of glutamic acid, proline, aspartic acid, threonine and serine (101). It has been reported that the mucin domains in the small intestinal of chicken are composed of proline, threonine and serine at approximately 22, 29 and 30% of the total amino acid sequences, respectively (44). A study conducted on broilers, laying hens and roosters has speculated that relative comparison of proline, glutamic acid, aspartic acid, serine and threonine concentration (as the main amino acids of mucoproteins) in the ileal digesta can be used for quantitative estimation of mucoproteins contribution (mucus loss) to the ileal endogenous amino acids loss of poultry (116). To our best knowledge, a specific method for measuring the contribution of mucoproteins to ileal endogenous amino acids in poultry has not been established yet, while it has been done for other species. In pig, the contribution of mucins in digesta was determined by the regression equation of the GalNAc : GluNAc ratio of purified mucin. The amount of mucins secreted into ileal digesta accounted for 13% by weight of which, 64% was gastric mucin and 36% was intestinal mucins (117, 118). Although endogenous loss of proteins and amino acids in chickens has been widely studied, the contribution of intestinal mucins in it has not been clearly explained. In the poultry studies that have considered this matter, the contribution of intestinal mucins in the endogenous amino acids’ loss has been attributed to the endogenous flow of the predominant mucin-derived amino acids.

## Alterations in Mucus Production by Diet and Feeding

Mucus production is associated closely with the digesta and gut movements as well as bacterial enzymatic digestion. Therefore, alterations in feeding strategies and diet e.g. feed restriction, protein level, carbohydrate sources, feed form, etc., affecting nutrient digestibility and gut bacterial status (e.g. symbiosis and dysbiosis) could potentially influence the intestinal GC as well as mucins production and dynamic.

Feed restriction in chicken has been shown to alter the number and secretion of GC in the small intestine. Feed restriction after hatch reduced cell proliferation and migration rate in the small intestine resulting in decreased number of enterocytes per villus, increased GC density and reduced villus surface area (2, 119). Moreover, during feed restriction (24-36 hours) in newly hatched chicks, a reduction in number of GC migrating from the crypt to the villus base has been reported, causing a lesser GC number in the lower half of villus in the jejunum and ileum compared with the upper part of villus (120). On the other hand, the expression of MUC2 mRNA in the small intestine was reduced in newly hatched chickens with delayed feed access up to 72 hours, which led a decrease in mucus production (7, 120). This may also be considered as an indication for immature GC or/and lower total GC number in the small intestine. It has been shown that feed restriction up to 36 hours suppressed proliferation and differentiation of stem cells therefore, the number of GC in the crypt did not change (increase) and cell migration out of the crypt decreased (120). The reason for the observed increase in GC density in the villi by feed restriction could be the reduction in villus surface area (not increase in GC number *per se*) or boosted host defense mechanism during the delayed development of gut barrier (121). The gut barrier and immune system are generally immature in newly hatched chicks therefore, the presence of mucus is of high importance for gut protection against the invasion of pathogenic bacteria and toxins (7). Immediate feeding of hatchlings is essential and required to support the development of intestinal epithelium including enterocytes and GC, in order to strengthen mucosal barrier to prevent damages by pathogens and toxins.

A few reports showed that delayed feeding in newly hatched chicks influences mucin composition in the GC. The proportion of acidic mucins increased in 48 hours fasted newly hatched chicks compared with fed chicks (7, 122). The presence of acidic mucins can have a protective role against bacterial invasion because of the fact that they are less prone to degradation by bacterial enzymes (63). Thus, the reported increase in acidic mucins may be associated with reported mucosal injury and bacterial overgrowth triggered by stress and decreased intestinal movement in fasted chickens (123). However, after having access to feed, the fasted chickens showed an ability to restore cell proliferation and their GC density. The cell proliferation and migration rate can be recovered within 3-4 days after refeeding (119). The GC density in these refed chicks was similar to immediately fed chicks during the first week of age (2, 120).

The effects of fasting on intestinal mucus layer has been investigated in older broiler chickens. Interestingly, the amount of mucus secreted per area of intestinal tissue of growing chickens (>4 weeks of age) was decreased with 72 hours feed restriction (45, 124), while the mucin concentrations (measured by the intensity of the bands using Western blot analysis) was increased (45). The decreased amount of mucus secreted per area might be resulted from an alteration in mucus composition especially reduction in water content, which also leads to a higher mucus concentration (125). It was proposed that the reduction in mucus secretion due to feed withdrawal in chicken may be associated with physiological regulation *via* cholinesterase activity (45). Mucus secretion is stimulated by acetylcholine. Feed restriction could increase acetylcholinesterase activity and subsequently decreases the stimulating signal for mucin secretion (30). In conclusion, the consequences of delayed feeding of newly hatched chicks and feed withdrawal for growing chickens is a reduction in differentiation and secretion of GC, leading to a thinner protective mucus layer and increased risk of exposure of the epithelium to luminal harmful agents. The restoration of GC population after feed introduction has been found at young age but the secretion ability of GC after feed introduction still needs to be studied further.

Dietary proteins and specific amino acids have been shown to alter mucin secretion through increasing renewal of mucus layer (126) or through providing amino acids essential for the mucin synthesis (127). Certain amino acids including threonine, proline and serine are of particular interest because of the role they play in the mucin amino acids backbone. Threonine is one of the essential amino acids which cannot be synthesized by poultry and must be provided by the diet. It was found that a reduction in dietary threonine by 60% and 30% of broiler requirement (8.2 g/kg diet) decreased crude mucin concentration in excreta by 50% and 20%. It also reduced sialic acid concentration by 49% and 9%, respectively (105). Similarly, feeding a low protein diet (19% CP) to broilers between 21 and 42 days of age, with a decreased level of threonine, proline and serine by 4, 18 and 17% respectively, caused a reduction in crude mucin excretion by 6.4-8.8% in compare with those received the standard (21% CP) diet (128). Therefore, mucin secretion may be disturbed by deficiency of some the amino acids like threonine.

Indigestible carbohydrates have an effect on luminal components including gut microbiota, gut epithelium and mucin secretion which was discussed by Montagne et al. (106). The indigestible carbohydrates including dietary fiber (DF) play a role in regulating mucus secretion due to their properties including water-holding capacity, viscosity and abrasive surface, which could potentially alter the quantity, physicochemical properties and protective function of the intestinal mucus layer (105). The secretion of intestinal mucins in rat was found to be proportionally increased with increasing the volume of insoluble DF attained in water (bulk-forming properties) (129) and with the viscosity of soluble DF (130). The stimulatory effects of DF on mucin secretion could be because of an increased luminal pressure and flow resistance of bulky and viscous digesta which enhance mucus loss and GC differentiation and subsequently, increase mucin secretion (130). In chicken, several studies showed that the addition of soluble or insoluble DF stimulated GC population and mucin secretion in the small intestine (121, 131). As an example, adding either insoluble (2-4% cellulose) or soluble fiber (2-4% carboxymethyl cellulose) to chicken diets increased ileal GC number compared with the control group (131). Similarly, the addition of insoluble DF compound (rice hull, 100 g/kg) enhanced MUC2 expression, increased number of GC per villus and increased mucin secretion in the jejunum and ileum compared with the control (cornstarch) diet (121). Different types of DF sources have also been shown to increase the excretion of mucins at the terminal ileum in pigs (e.g. peas, wheat, straw, corn cobs and cellulose), rats (psyllium seed husk) and human (soya fiber) (106).

Different cereal types provide varied composition and amount of DF which could modify bacterial fermentation and their metabolic activities (132). Non-starch polysaccharides (NSP) are of high importance since they are largely indigestible in the small intestine of poultry and are mainly fermented in the hindgut by bacteria (106, 133). Cereals with high soluble NSP content like wheat, barley, rye and oat, can lead to a high viscous conditions in the small intestine and may alter the intestinal bacterial composition and activities compared with cereals like corn which contains lower soluble NSP (79, 134). The main fermentation by-products of NSP are short chain fatty acids (SCFA), predominantly acetate, propionate and butyrate. Approximately 95 to 99% of SCFA that are produced in the hindgut of non-ruminants, are absorbed and have specific roles in the body (135). As for example, acetate which is the most abundant SCFA, acts as an energy substrate for muscle tissue and can be utilized by bacteria as a precursor for butyrate synthesis. Propionate regulates glucose synthesis in liver and butyrate is used as a major source of energy for cellular metabolic activities (132). It has been shown *in vitro* and rat studies that SCFA, in particular butyrate, also involve in supplying energy for intestinal GC proliferation and differentiation and subsequently increase mucus production and MUC2 gene expression in the gut (136, 137). Feeding broilers with wheat/rye-based diet instead of corn-based diet increased bacterial number in the small intestine, most notably enterobacteria, and increased SCFA concentration, especially acetate and n-butyrate in the cecum (133). It also increased GC size and their number in the ileum and the cecum (134). Although soluble NSP provide the energy for bacteria which allow them to use other nutrients such as nitrogen as substrates for metabolite production, it should be concerned that the presence of these viscous-forming fibers have adverse effects on nutrient absorption. The viscous NSP can physically complex with intestinal enzymes reducing the interaction with substrates, thus decrease nutrient digestibility (133). Using SCFA as feed additives for poultry has been shown to promote intestinal development and modulate gut bacteria (138). Adding sodium butyrate (0.8 g/kg) to broiler diets caused a distinct impact on the bacterial community and increased the number of bacteria related to the fermentation of undigested carbohydrate including *Firmicutes, Bacteroidetes* and *Proteobacteria* in the cecum and thus, further increased microbial-derived SCFA compared with the control group (138). Addition of sodium butyrate (0.2-1 g/kg) in broiler diets increased villus length and GC density in the jejunum and ileum, and also increased mucus secretion compared with the control diet (138). Supplementing sodium butyrate (0.5-1 g/kg) in broiler diets also increased acidic GC number per villi of the small intestine, suggesting a stimulating effect for butyrate to promote protective mechanism against mucin degradation by gut bacteria (9). In conclusion, the recent obtained data shows a potential role for dietary and bacterial SCFA specially butyrate, in regulating GC differentiation and modulating mucus production and dynamic.

The oxidative stress of the intestinal cells (e.g. colonic GC) induced by high fat diet, led to upregulation of intestinal inflammatory cytokines (e.g. IL-1b, TNF-α and IL-17a), along with a decrease in GC differentiation and MUC2 expression in mice (139). There is a limited information available regarding the impact of dietary fat on mucus properties in poultry. However, the intracellular fatty acid-binding proteins (FABPs) have been subject of several studies. In poultry, FABPs modulate lipid metabolism via regulation in the fatty acid uptake (in line with the concentration gradient) into the cell (140). Several FABPs including FABP1, FABP2, and FABP6 have been identified to be predominantly expressed in the digestive tract of chickens (140, 141). It has been shown that, enhancing the dietary fat level in poultry feed could increase the concentration of FABPs in the intestine (142). A downregulation in mRNA expression of FABP2 occurred in compromised gut barrier chickens (challenged with coccidiosis vaccine) along with decreased MUC2 and occludin expression, which may indicate an association between FABP2 and gut integrity in chickens (141). It was suggested that necrotic enteritis infection in broilers caused downregulation of FABP1 and FABP2 in the small intestine. These downregulations were assumed to be attributed to structural damage and intestinal epithelium loss in the small intestine and lead to reduction in fatty acid utilization (140). However, to the best of our knowledge, no direct interaction between FABPs and mucus production and/or quality has been reported so far.A downregulation in mRNA expression of FABP2 occurred in compromised gut barrier chickens (challenged with coccidiosis vaccine) along with decreased MUC2 and occludin expression, which indicate the role of FABP2 in maintaining intestinal integrity in chicken (141). It was suggested that the downregulation of FABP1, FABP2 and other genes that are related to reduced fatty acid utilization may be also associated with intestinal inflammation and structural damage of the epithelium (140). The concentration of lipids in poultry feed is considerably lower than carbohydrates and proteins, but they still influence gut microbiota (143, 144). Feeding isoenergetic diets with different fat sources to broilers affected the pH and fermentation products in the ileum and cecum, which shows differences in activity and composition of the gut microbiota between these groups (144). For example, diet with palm kernel fatty acids distillers (4.3%) increased concentration of total SCFA and lactate in the ileum and cecum and decreased digesta pH in the ileum compared with soybean oil (4.0%) diet (144). As mentioned above, higher SCFA production in the gut can lead to higher GC proliferation and differentiation as well as higher mucus production (136, 137). Therefore, dietary fat level and type in poultry feed can have an indirect impact on intestinal integrity and mucus production. However, the extent of this impact needs further investigation.

## Conclusion and Suggestions

It can be concluded that the intestinal mucus layer plays an important role in maintaining the intestinal microbial balance, facilitating nutrient transport, preventing pathogen invasion, and regulating the microbial–host immune response. The intestinal mucus layer made by mucins secreted by goblet cells possesses a particular structure and molecular glycan composition for each part of the gut which contributes to its main functions including protecting itself against sheer force of dietary materials, transporting nutrient, maintaining the colonization of commensal bacteria and protecting the epithelial surfaces against pathogenic bacteria. In chicken, the considerable increase in the intestinal goblet cells density and activity in the first week of age is a response to emerging needs of newly hatched chickens for mucus secretion and immune response associated with their immediate expose to the surrounding environment and diet. The goblet cell population in the small intestine of chickens reaches maturity at 3 weeks of age. There is an anteroposterior increasing trend in the goblet cells density and mucus secretion in the small intestine of chickens which is a host adaptation to enhance protective barrier against the increasing number (and activity) of gut bacteria. Furthermore, the proximal part of the small intestine including duodenum is very active in digestion and absorption and may prioritize the proliferation of absorptive cells over goblet cells, which is associated with a lower goblet cell density, lower mucus secretion and a larger goblet cell size in the duodenum compared with the jejunum and ileum. The continuous production of mucins by goblet cells mainly renews the outer mucus layer which is easily lost into the lumen by mechanical erosion and bacterial degradation. However, the regulated secretion of mucus is a rapid response to external stimuli and acts as the first defensive mechanism of the gut. The distribution of mucin types in the goblet cells is regulated by glycosylation of *O*-glycan, which can be affected by the host (e.g. inflammatory markers, hormones and neurotransmitters) and external (e.g. commensal bacteria, pathogens, pre/probiotics and nutrients in the diet) factors. Increasing in acidic mucins is known as an adaptation strategy to protect mucin from degradation by bacteria. However, several bacteria have an ability to still degrade this barrier. Any factor affecting nutrient digestibility, gut motility and digesta flow, gut bacterial status and their metabolic activity e.g. dietary factors (physical and chemical properties of feed) could potentially influence the intestinal goblet cells as well as mucin production and dynamic. The mode of action and mechanisms behind these effects need to be studied further. Mucins resist to digestive processes; therefore, a significant fraction of endogenous losses in chicken is mucins which can be considered as a loss of protein and energy to animal. Hydrothermal processing of poultry feed may reduce this loss by reduction in mucus shedding into the lumen. Given the significance of this loss and the lack of precise data about it, this matter needs to be carefully investigated in the future and the nutritional strategies reducing this loss have to be defined better.

## Author Contributions

Conceptualization, YD, JZ, and FGB. Writing-Original draft, YD. Writing-Review and Editing, YD, JZ, and FGB. All authors contributed to the article and approved the submitted version.

## Conflict of Interest

The authors declare that the research was conducted in the absence of any commercial or financial relationships that could be construed as a potential conflict of interest.

## Publisher’s Note

All claims expressed in this article are solely those of the authors and do not necessarily represent those of their affiliated organizations, or those of the publisher, the editors and the reviewers. Any product that may be evaluated in this article, or claim that may be made by its manufacturer, is not guaranteed or endorsed by the publisher.
